# Serotyping and Seroprevalence of *Mannheimia haemolytica, Pasteurella multocida*, and *Bibersteinia trehalosi* and Assessment of Determinants of Ovine Pasteurellosis in West Amhara Sub-region, Ethiopia

**DOI:** 10.3389/fvets.2022.866206

**Published:** 2022-05-19

**Authors:** Kalkidan Getnet, Bezawit Abera, Haymanot Getie, Wassie Molla, Sefinew Alemu Mekonnen, Bemrew Admassu Megistu, Anmaw Shite Abat, Haileyesus Dejene, Mastewal Birhan, Saddam Mohammed Ibrahim

**Affiliations:** ^1^Department of Veterinary Epidemiology and Public Health, University of Gondar, Gondar, Ethiopia; ^2^Department of Veterinary Biomedical Sciences, University of Gondar, Gondar, Ethiopia; ^3^Department of Veterinary Paraclinical Studies, University of Gondar, Gondar, Ethiopia

**Keywords:** *B. trehalosi*, indirect haemagglutination, *M. haemolytica*, ovine pasteurellosis, *P. multocida*, predisposing factors, seroprevalence

## Abstract

A cross-sectional study was undertaken in four (4) districts of the West Amhara sub-region of Ethiopia with the aim of assessing the diversity and distribution of serotypes of Pasteurella species, their seroprevalence, and associated risk factors, and knowledge, attitude, and practice of farmers toward ovine pasteurellosis. A total of 600 sheep sera were collected using multistage cluster sampling. Each sample was examined for the presence of six (6) serotype-specific antibodies using an indirect haemagglutination test. We are reporting a higher seroprevalence of 90.17% (541/600) in which all seropositive animals were shown to have been co-infected with multiple serotypes. Individual serotype prevalence showed that serotype A7 has the highest prevalence of 77.83% followed by A2 (74.33%), T15 (64%), T4 (62%), PA (60%), and A1 (39.17%). In this study, being female [odds ratio (OR): 2.45, 95% CI (1.09–5.52), *p* = 0.031] and living in high altitude areas [OR: 20.29, 95% CI (2.54–161.95), *p* = 0.004] were found to be significantly associated with sero-positivity. A questionnaire survey (*n* = 384) employed in a face-to-face interview was used to assess the knowledge, attitude, and practice of farmers related to ovine pasteurellosis. Accordingly, the majority (72.4%) of respondents had an inadequate knowledge level of the disease. The proportion of farmers with a favorable attitude and good practices toward the disease was 50.26 and 77.6%, respectively. This study is highly indicative that ovine pasteurellosis is a ubiquitous disease in the study area challenging the sheep production sector. The existence of diverse serotypes reported to lack cross-protective immunity is likely to explain why the current vaccination practice with the mono-serotype *Pasteurella multocida* biotype A vaccine is not providing adequate protection against outbreaks of the disease. Prioritization of one or more serotypes for inclusion in a multivalent vaccine should be dictated by the abundance and distribution of a particular serotype, its clinical importance, and its resultant economic impact. Furthermore, training farmers on key aspects of the disease is vital in the implementation of effective disease management strategies through a participatory approach. Data from the remaining regions of the country could help realize the development of an effective vaccine that works best at the national level.

## Introduction

Sheep production is a key agricultural activity throughout Ethiopia, a country with an enormous sheep population of about 43 million by the year 2020 (1). Several attributes such as adaptability to harsh environmental conditions, less feed and space requirement, high fertility, short reproductive interval, high off-take rate, and low initial investment cost make this segment of livestock a viable option for resource-limited farmers in the country. Sheep are multiple purpose animals offering their owners a wide range of products and services such as meat, milk, skin, hair, wool, bones, manure, security, gifts, and religious rituals (2, 3). Currently, the Ethiopian population is growing at an alarming rate potentially worsening the already existing food insecurity in the country (4). Small ruminant production is vital in ensuring food security (5).

According to investigations in household surveys in Ethiopia, infectious diseases are consistently reported to be the major hurdles constraining smallholder livestock activities (6). Owing to its substantial morbidity and mortality (7, 8), pasteurellosis is ranked as one of the priority diseases threatening sheep production in various regions of the country (6). This is corroborated by various reports of the disease in different parts of Ethiopia (9–13) which underscored the need to understand the epidemiology of the disease and devise effective control strategies. In addition, Ayelet et al. (2004) (9) in their year-long longitudinal study on multifactorial respiratory diseases of sheep, concluded that pneumonic pasteurellosis is a disease of concern to sheep production as well as smallholder farmers.

The etiological agents for ovine pasteurellosis are *Pasteurella multocida, Mannheimia hemolytica*, and *Bibersteinia trehalosi*, normal inhabitants of the tonsil and oropharynx of healthy animals. The disease is always initiated when animals are exposed to environmental stressors such as adverse weather (hot or cold), inadequate ventilation, shipping, feed/water shortage, and concurrent infection (14). A remarkable feature of these bacterial agents is the presence of abundant serotypes of clinical and immunological significance (15–17). Based on lipopolysaccharide antigens, *P. multocida*, can be classified into 16 serotypes which can be regrouped into five (5) serogroups (A, B, D, E, and F) based on passive haemagglutination testing of capsular antigens (18). Several works on *P. haemolytica* lend to the naming of *M. haemolytica* and *B. trehalosi* (19). First*, P. haemolytica* was classified into two (2) biotypes, A and T, depending on the tendency to ferment arabinose and trehalose, respectively (19). Further re-classification of these biotypes based on their capsular antigens by indirect haemagglutination (IHA) test resulted in 17 serotypes, i.e., A1, A2, A5–A9, A11–A14, A16, and A17 belonging to the *P. haemolytica* biotype A, and T3, T4, T10, and T15 belonging to *P. haemolytica* biotype T. Later, *P. haemolytica* biotype A and *P. haemolytica* biotype T was renamed as *M. haemolytica* and *B. trehalosi*, respectively (20). The classification gets broader with the re-categorization of serotype A11 (due to its unique biochemical features) into a new genus, *M. glucosida* (21). Therefore, *M. haemolytica, B. trehalosi, and M. glucosida* represent a collection of 12, 4, and 1 (1) serotypes, respectively. It's worth noting that there are also reports of additional Non-typeable isolates of *M. haemolytica* further depicting the complexity of these pathogens (17).

Identification of serotypes and their distribution may serve various purposes. Clinical, postmortem, and microbiological evaluation endpoints have shown that different serotypes have varying degrees of virulence and pathogenicity. In addition, they exhibited variations in host preference and adaptability with potential inter-species transmissibility, antigenicity, immunogenicity, and drug resistance pattern potentially impacting the course of the disease as well as a choice of treatment and its outcome (22). Of particular concern to the management of the disease through vaccination, is the lack of cross-protection among the serotypes, i.e., immunity against a given serotype may provide no benefit against infection with other serotypes (23–25). Despite this, the status of circulating serotypes of *M. haemolytica, P. multocida*, and *B. trehalosi* in the sheep population in the Amhara region remains largely unknown. Nowadays, a vaccine based on killed *P. multocida* biotype A (NVI, Bishoftu, Ethiopia) coupled with other management strategies is used on farms throughout Ethiopia to help control the disease, and avoid its drastic consequences (26). However, ovine pasteurellosis remains a major concern with several reports of cases and outbreaks in the Amhara region. A mismatch between the circulating field and vaccinal serotypes is a critical factor impacting vaccine effectiveness particularly when cross-immunity is partial or Non-existent among the different serotypes (27). The other important factor that facilitates the success of control of the disease is farmer/community engagement through a participatory approach (28) which in turn is influenced by the level of knowledge, attitude, and practices (KAP) of farmers toward the disease. Therefore, in this study, we aimed to determine the diversity and distribution of circulating serotypes of *M. haemolytica, P. multocida*, and *B. trehalosi*, their prevalence, the predisposing factors, and the KAP of farmers toward ovine pasteurellosis.

## Materials and Methods

### Study Area

**Figure 1:**
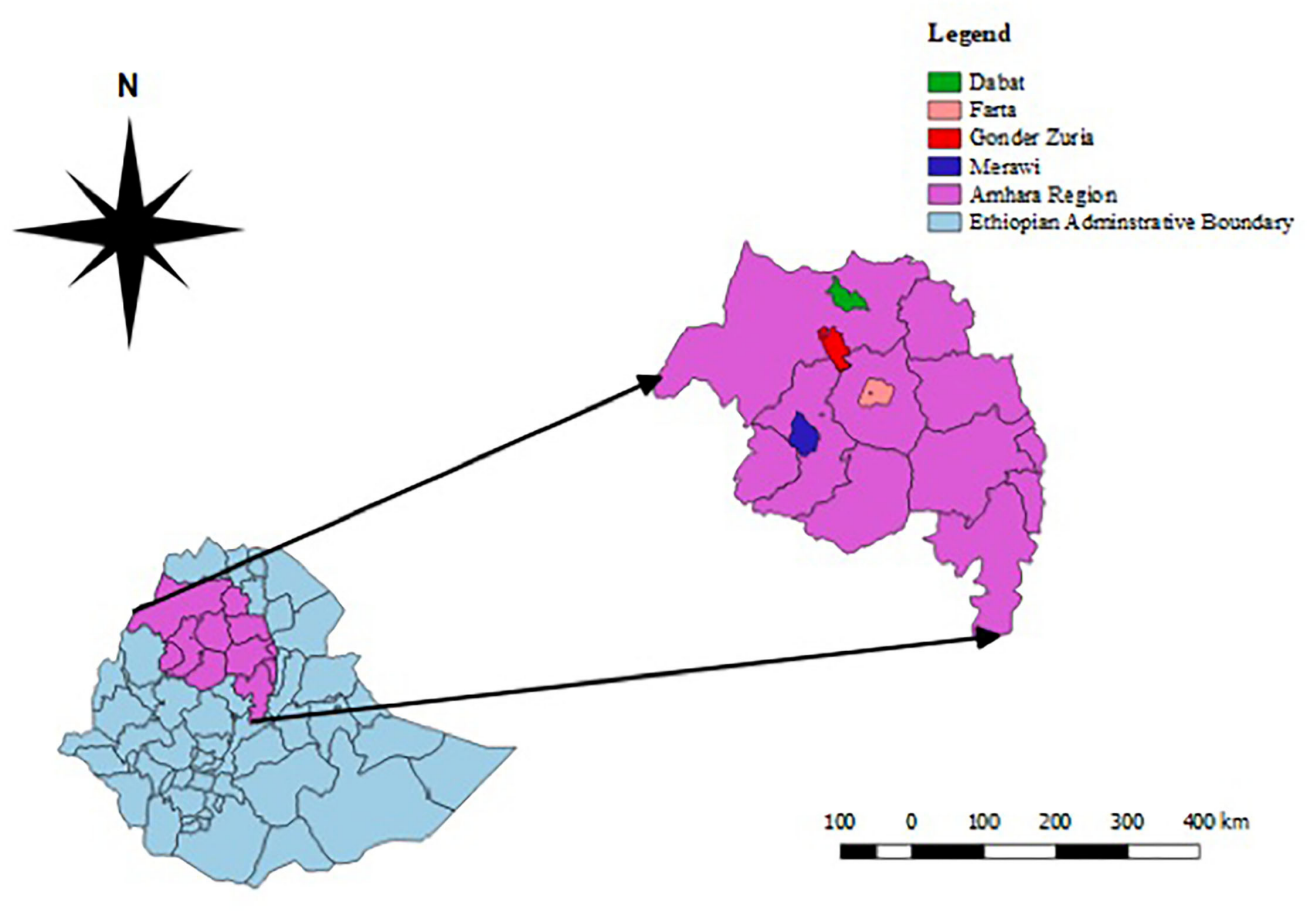
Map depicting the different districts of the study area in the West Amhara sub-region. The map was created by QGIS version 2.18.1 Las Palmas.

This study was carried out in selected zones of the Western Amhara sub-region, namely, West Gojam, Central Gondar, South Gondar, and North Gondar zones located in Northwest Ethiopia (Figure 1). The sub-region is situated between 10–14° North latitude and 35.1–38.35° East longitude with a total annual rainfall ranging from 878 mm to 2,100 mm, and annual average maximum and minimum temperature of 30.7 and 22°C, respectively. The area is characterized by different agro-climates with subsistence crop-livestock production systems (29) and ranked first in its small ruminant population in the Amhara regional state (1). A significant number of people live through agricultural activities including small-scale farming, supplying food animals, and animal products to the communities and slaughterhouses. Amhara region shares extensive internal boundaries with Benshangul Gumuz, Oromia, and Tigray regional states of Ethiopia, and is bordered by Sudan where transboundary livestock movement is common.

### Study Design and Study Animals

A cross-sectional study design was used to collect blood samples and questionnaire data from October 2019 to June 2021. The study animals were indigenous breeds of sheep kept under an extensive, semi-intensive, and intensive management system. Sheep were considered for sampling when they were found above 4 months of age and with no history of vaccination against pasteurellosis.

### Sampling Technique

Multi-stage cluster sampling method was employed, in which four (4) districts from the Western Amhara sub-region were considered. The selection of districts was based on representativeness in terms of agroecology, an abundance of sheep population, and prior complaints of respiratory diseases. Kebeles (lowest administrative divisions) and villages (sub-divisions of a kebele), and individual animals were selected using simple random and systematic sampling, respectively. Three (3) kebeles from each of two (2) districts and two (2) kebeles from the other two (2) districts were selected based on the proportion of kebeles within the districts. Kebeles with a history of vaccination were excluded from the sampling frame. Finally, three (3) villages from two (2) kebeles and two (2) villages from the remaining eight (8) kebeles were selected proportional to the number of villages available. In this study clusters were villages. Thus, blood samples were collected from a total of 22 villages.

### Sample Size Determination

The sample size for the sero-epidemiology was determined using the formula described by Bennett et al. (30).


$$\begin{array}{l} n=gc=\frac{P\left(1-P\right)D}{SE^{2}} \end{array}$$

Where “*n*” is the sample size, “*p”* is the prevalence as a percentage, “*D”* is the design effect, “*SE*” is the standard error, “*g”* is the average number of individuals sampled per cluster, and “*c”* is the number of clusters.


$$\begin{array}{l} D=1+\left(g-1\right)ICC \end{array}$$

The estimate of intracluster correlation coefficient (ICC) for most infectious diseases does not exceed 0.2 (31). So, taking 0.2 for the cluster (village) and considering the possibility to collect about 30 serum samples (g) in a village, D equals 6.8. Sampling 30 animals per village with an expected prevalence of 50% (as no previous studies were conducted in the study area) and a standard error of 0.05 gave about 22 clusters, and thus a total sample size of 680 animals. However, the total number of samples collected was 600, due to owners' consent during sampling. The clusters were proportionally distributed among 4 districts and 10 kebeles, i.e., 7 clusters (from 6 kebeles) were considered from each of the two districts having 41 and 44 kebeles (Merawi and Gondar zuria districts), and 4 clusters (from 4 kebeles) from each of the other two districts having 32 and 34 kebeles (Farta and Dabat districts).

For the questionnaire survey, sheep owners who consented to participate were recruited for the interview. The sample size was calculated based on the formula described previously (32).


$$\begin{array}{l} n=\left(t\right)2⋇\left(p\right)\left(q\right)/\left(d\right)2 \end{array}$$

Where “*t”* is the value for a selected alpha level of 0.025 in each tail which equals 1.96, “*p”* is the proportion of the population that had knowledge about the disease which is 0.5, “*q”* proportion of the household who did not have knowledge about the disease which is 0.5, “*(p)(q)*” is an estimate of variance across the population, and “*d”* the acceptable margin of error which is equal to 0.05. Thus, 384 households were interviewed.

## Data Collection Methods

### Blood Sample Collection and Laboratory Analysis

About 8 ml of blood was collected aseptically from the jugular vein of each animal using a plain vacutainer tube. Blood containing tubes were kept horizontally and rendered to clot without agitation for 2 h at room temperature, then samples were transported in an icebox to the microbiology laboratory of the college of veterinary medicine and animal sciences at the University of Gondar, where they were placed tilted overnight at 4°C. Serum was then separated from the clot by centrifugation at 3,000 rpm for 10 min, transferred into labeled cryovials, and kept frozen at −20°C until shipment to the National Veterinary Institute (NVI), Bishoftu, Ethiopia for serotyping. An indirect haemagglutination (IHA) test was performed to determine the presence of serotypes-specific antibody reactions as per the methods described by (33, 34). The presence of a dense clot at the bottom of the microtiter well was considered a positive agglutination reaction while the presence of uniform suspension of the red blood cells indicated a negative reaction. An agglutination rate of >50% was taken as positive (35), and those showing a hemagglutination reaction in 1/20 dilution and above were taken as positive samples (9). Each sample was tested for six (6) different serotypes, namely, A1, A2, A7 of *M. haemolytica*; T4 and T15 of *B. trehalosi;* and biotype A of *P. multocida*. Controls used were sera containing high titer of antibodies against each serotype (positive controls) and sera with no antibodies (negative controls), all obtained from NVI, Bishoftu, Ethiopia.

### Questionnaire Survey

A semi-structured questionnaire was used to collect information on the KAP of sheep owners and the predisposing factors favoring the occurrence of the disease in the study areas. The questionnaire comprised three (3) parts: part-I, designed to collect socio-demographic information about the respondents; part-II, intended to assess predisposing factors for the disease; and part-III, purposed to assess the KAP of farmers toward ovine pasteurellosis (Supplementary Material 1). The data were collected through face-to-face interviews using the local language (Amharic). Before starting an actual data collection, the questionnaire was pretested to identify gaps. When assessing KAP, the disease's local name “*Gororsa”* was used to communicate with farmers, and only those aware of it were considered for the whole interview.

### Assessment of Predisposing Factors

Data relevant to evaluating risk factors for the occurrence of ovine pasteurellosis were collected along with blood specimens. The individual animal details such as sex and age were recorded. Disease determinants like flock size, presence/absence of concurrent infection, contact with other flocks, husbandry system, and agro-climatic zones (such as midland and highland) were registered. The ages of individual sheep were estimated according to Abegaz et al. (36) and were categorized into three age-groups: <1 year (young), 1–2 years (adult), and >2 years (old). Regarding geographical attributes, areas with an altitude <1,500 m.a.s.l were categorized as lowland while areas ranging from 1,500 to 2,300 m.a.s.l and >2,300 m.a.s.l as midland and highland, respectively. Sheep flock sizes were categorized as a flock of <5 sheep, 5–10 sheep, and >10 sheep. In addition, location (zones, districts, kebeles, and villages), name of household, and global positioning system point (longitude and latitude altitude) data were taken from the sampling area.

### Assessment of Knowledge, Attitude, and Practices of Farmers

Knowledge was assessed using four (4) questions, two (2) of which were answered as “yes” or “no”, and the remaining two (2) asked farmers to list clinical signs related to the disease, and environmental stressors driving the occurrence of ovine pasteurellosis. The maximum attainable knowledge score was 11 (maximum of one point for each of Q#1 and Q#3, 5 points for Q#2, and 4 points for Q#4) while the minimum score was 0, by giving a score of 1 for correctly answered and 0 for wrongly answered questions. As the knowledge score of respondents was normally distributed, the mean is a good central value, which was then taken as a cut-off value for knowledge level. The knowledge level of respondents was categorized as “good” if the knowledge score was greater or equal to the knowledge mean score and as “poor” if the score was less than the mean knowledge score. The total sum of the knowledge scores was changed into binary outcome as adequate knowledge (for knowledge score ≥50%) and inadequate knowledge (for knowledge score <50%).

The attitude was assessed using five (5) questions developed as Likert's scale having five components valued as 1 = strongly agree, 2 = agree, 3 = neither agree nor disagree, 4 = disagree, and 5 = strongly disagree (37). As the attitude score was not normally distributed, the median is a good central value, which was then taken as a cut-off value for attitude delineation. Respondents who had scored points greater than or equal to the attitude median score were considered as having desirable/favorable attitudes and those who did score less than the median were considered as having undesirable/unfavorable attitudes toward the disease (38).

The practice was assessed using five (5) questions answered as “yes” or “no” (by scoring 1 for correct answers and 0 for incorrect answers). The maximum and minimum attainable practice scores were 5 and 0, respectively. Practice score was normally distributed and thus mean a good central value which was then taken as a cut-off value for practice level. Respondents who scored greater or equal to the mean practice score were classified as having “good practice” and those who scored below the median practice score were considered as having “poor practice”.

### Data Management and Analysis

Raw data collected were entered into a Microsoft Excel spreadsheet (Microsoft Excel 2010, Microsoft Corporate, USA) coded, and then imported into STATA version 14 (Stata Corp., College Station, TX, USA) for statistical analysis. Descriptive statistics involving frequency and percentage were computed to illustrate the characteristics of the study population, KAP of respondents, and the seroprevalence and serotypes distribution in the study areas. Three separate multivariable models were developed to investigate the association of KAP with the socio-demographic characteristics of respondents. Initially, the cumulative score obtained for questions pertaining to the three response criteria (knowledge, attitudes, and practices related to ovine pasteurellosis) was converted into binomial outcomes by categorizing the respondents as having scored above or below the mean or median score for each response criteria. Binary logistic regression was performed with a 95% CI to determine associations between categorical dependent and independent variables. All explanatory variables with a *p* < 0.25 were included in the multivariable logistic regression models. The reduced subset models were developed using backward elimination based on the AIC (Akaike Information Criteria) score for each model. The final multivariable logistic regression models were evaluated using Pearson's and Deviance residuals and their goodness-of-fit was assessed by the Hosmer-Lemeshow test. Variables with *p* < 0.05 were retained in the final model.

For analysis of risk factors associated with the sero-positivity, mixed-effect logistic regression was performed. First, the potential risk factor variables were screened using univariable analysis, and then variables with *p* < 0.25 were subjected to multivariable analysis considering village as a random effect. Those variables screened for the multivariable analyses were checked for co-linearity and none were found collinear. A factor or independent variable of interest was considered to have an influence on sero-positivity at a significance level of below 5%. Nested models were performed by the use of the forward addition method and changes in the beta value (coefficient) of risk factor variables were computed to detect the presence of a confounder. If the change in the beta value of a certain risk factor variable following the addition of a Non-significant variable to the nested model is ≥30%, the added variable is considered a confounder and should be included in the final model. Animal level apparent prevalence was calculated based on sampled population by percentage as:


$$\begin{array}{l} Animal\;level\;Apparent\;prevalence\\ =\;{\frac{number\;of\;positive\;sample}{numbe\;of\;total\;sampled\;population}}^{*}100 \end{array}$$

## Results

### Seroprevalence of Ovine Pastuerellosis

**Figure 2:**
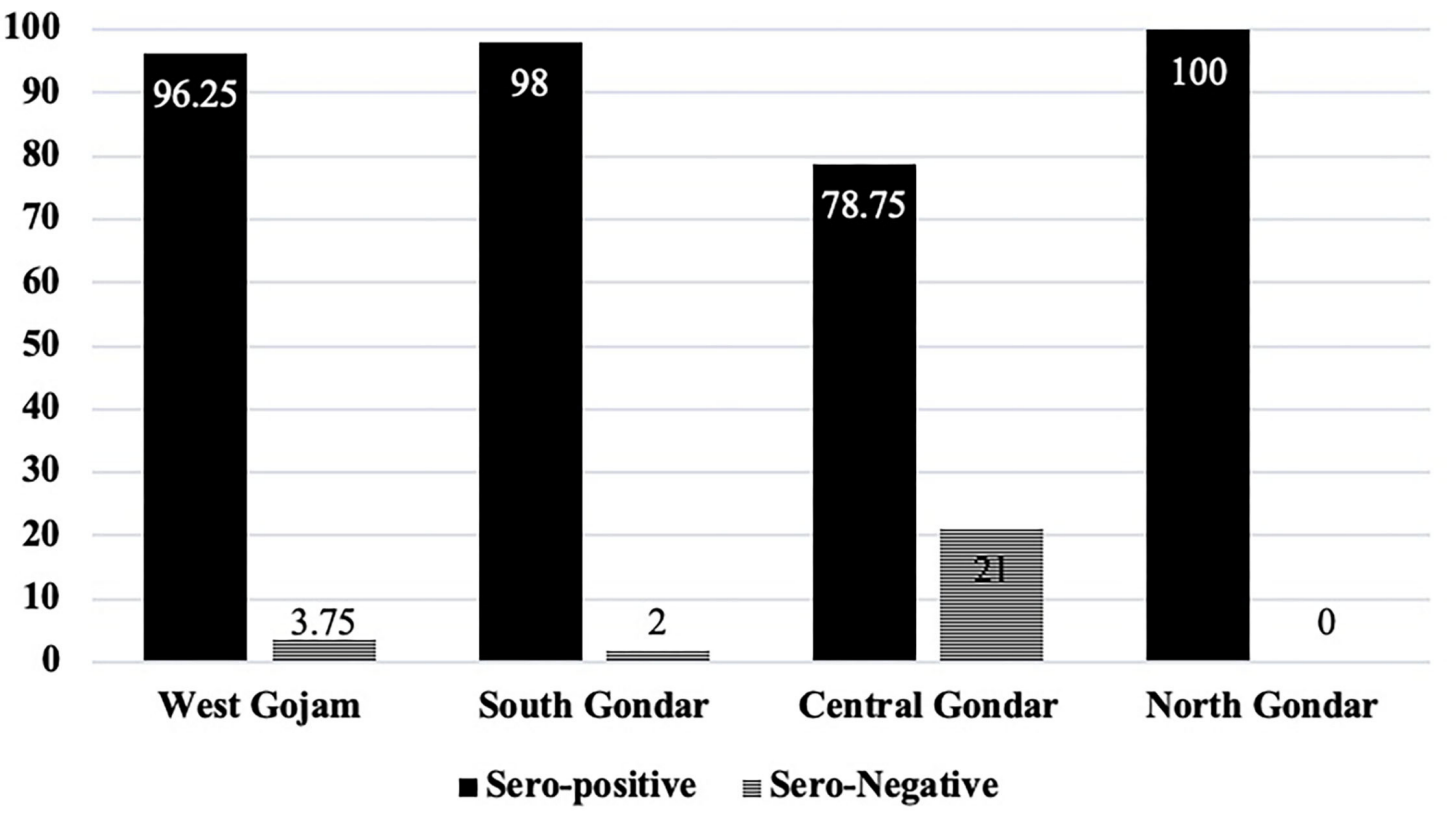
Seroprevalence (%) of ovine pasteurellosis in the four zones of West Amhara region. Serum was obtained from those zone as followed, West Gojam (*n* = 160), South Gondar (*n* = 100), Central Gondar (*n* = 240), and North Gondar (*n* = 100). IHA test was performed in each serum to determine the presence of serotype-specific antibody reactions. An animal is considered sero-positive if it tested positive for at least one of the six (6) serotypes examined (A1, A2, A7, T4, T15, and PA). The district-level prevalence was 96.25% (West Gojam), 98% (South Gondar), 78.75% (Central Gondar), and 100% (North Gondar) (*p* = 0). PA = *Pasteurella multocida biotype A*.

Of all sheep sampled (*n* = 600), 541 [90.17%, (95% CI: 88–93)] tested positive for at least one serotype of *M. haemolytica, P. multocida*, or *B. trehalosi*. The highest individual animal level apparent prevalence was recorded in North Gondar zone, Dabat district (100%) followed by South Gondar zone, Farta district (98%), West Gojam zone, Merawi district (96.25%) and Central Gondar zone, Gondar zuria district (78.75%) (Figure 2). Seroprevalence was shown to vary significantly among districts (*p* = 0.001).

#### Serotyping

**Figure 3:**
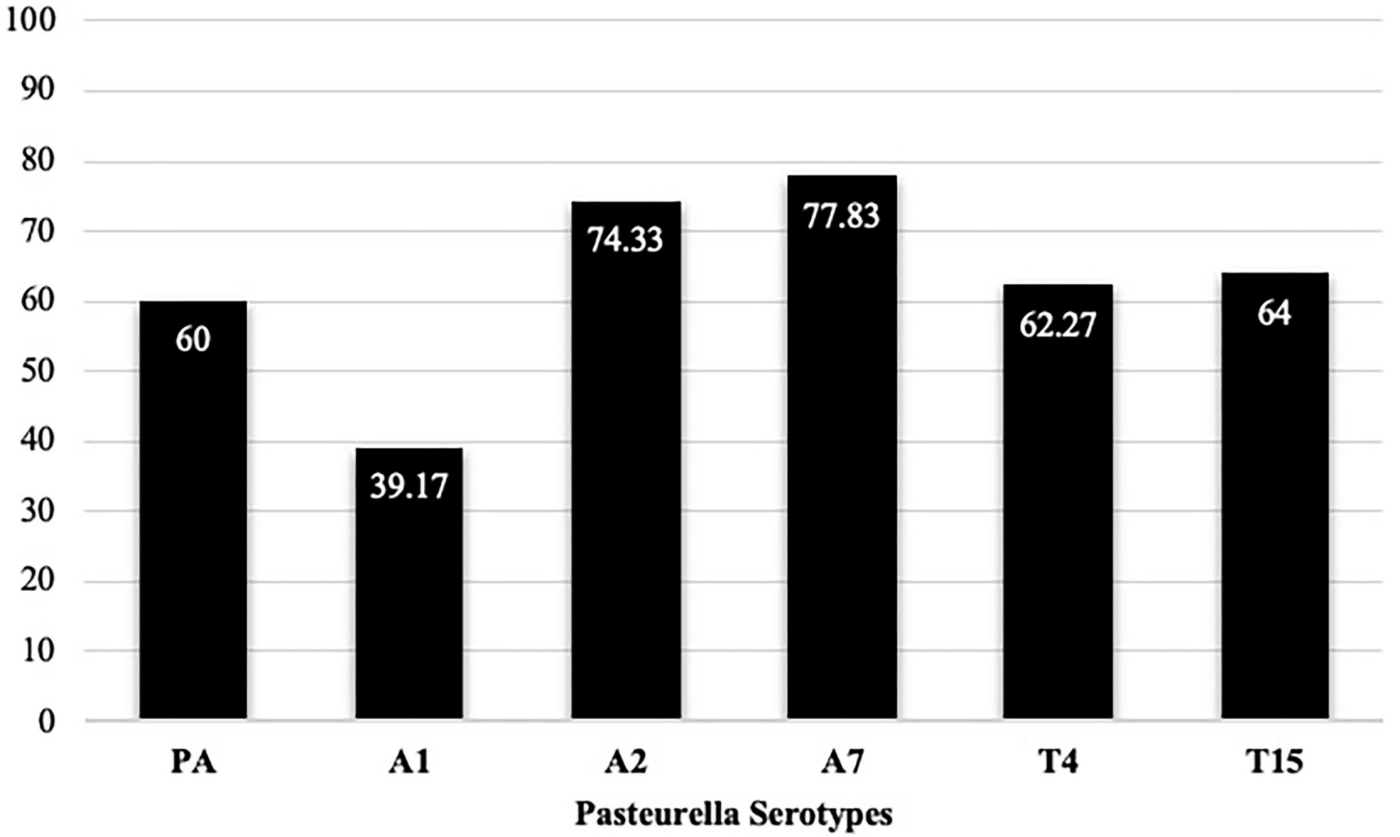
Individual-level serotype prevalence, in which 600 serum samples were collected from the study areas and each sample was subjected to IHA testing against six (6) serotypes. PA = *P. multocida biotype A*. Percentage of positive samples out of 600 is provided.

**Figure 4:**
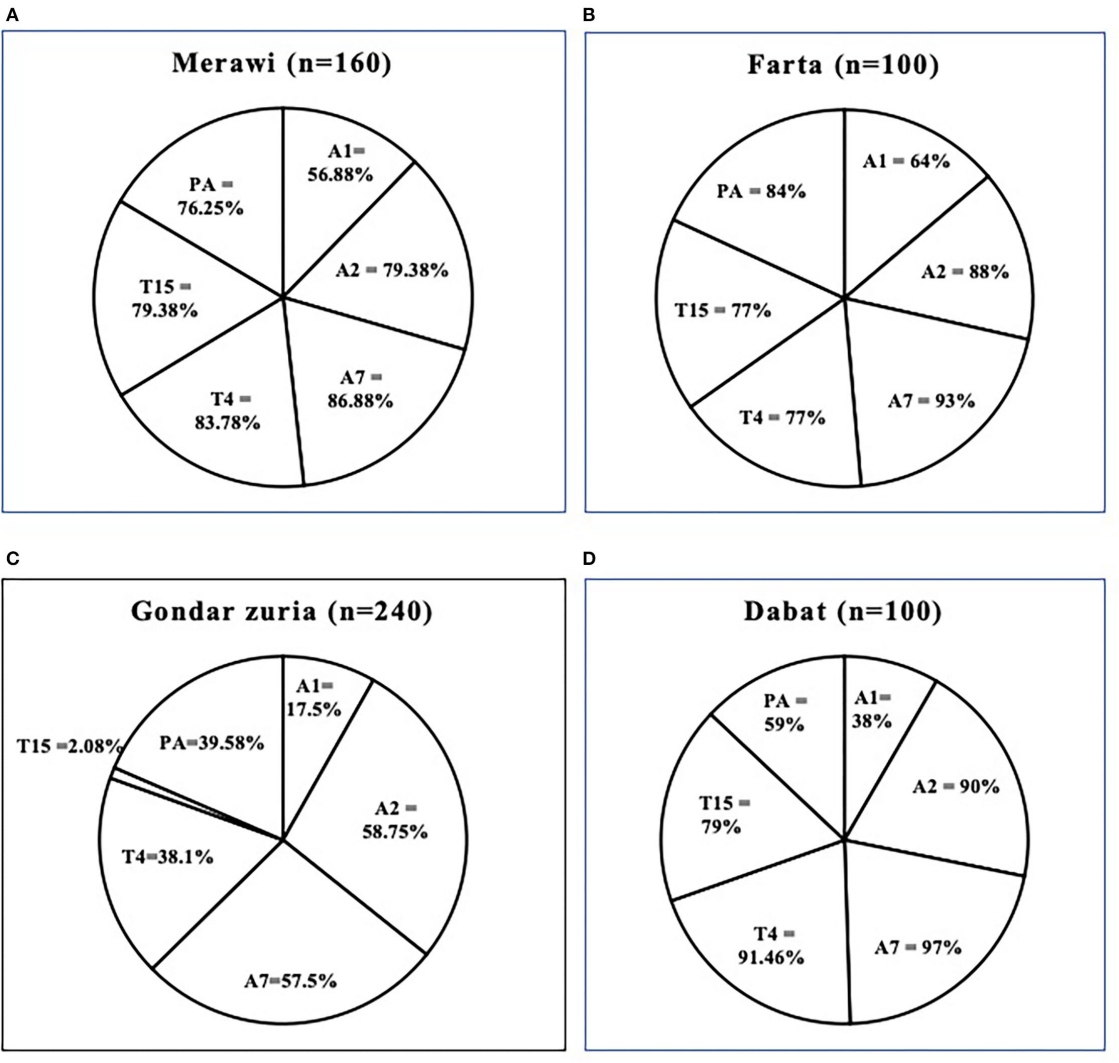
Prevalence and percentage of various serotypes of *M. haemolytica, P. multocida*, and *B. trehalosi* for each district: Merawi **(A)**, Farta **(B)**, Gondar Zuria **(C)**, and Dabat **(D)**. All the districts have been shown to have a high prevalence of the six (6) serotypes except for the Gondar Zuria district where T15 had an unusually low prevalence as compared to the other districts. Infection with multiple serotypes is apparent in all the sampled animals. PA = *P. multocida biotype A*.

As mentioned in the method, each serum sample was tested against six (6) serotypes using the IHA test i.e., A1, A2, A7 of *M. haemolytica; P. multocida A;* and T4 and T15 of *B. trehalosi*. Of all, the prevalence of A7 and A2 were the highest (77.83 and 74.33%, respectively), followed by T15 (64%), T4 (62%), *P*. *multocida* A (60%), and A1 (39.17%) (Figure 3). The district-level prevalence of each serotype is indicated in Figure 4.

### Risk Factors Associated With Ovine Pasteurellosis

Host and environment-related factors like sex, age, and altitude were included in the multivariable model (*p* < 0.25). The results of the univariable mixed-effect logistic regression model are provided in Table 1. The final model showed that female sex [OR: 2.45, 95% CI (1.09–5.52), *p* = 0.031] and higher altitude [OR: 20.29, 95% CI (2.54–161.95), *p* = 0.004] had a statistically significant association with sero-positivity while there was no interaction effect among sex and altitude (Table 2). The remaining factors appeared Non-significant in the multivariable analysis and had no confounding effect hence omitted from the final model.

**Table 1:** Respondents' knowledge level on ovine pasteurellosis (*n* = 384).

**Knowledge queries**	**Responses**	**Frequency**	**Percentage**
**Q#1**. Have you ever heard of ovine pasteurellosis (“*Gororsa”*)	Yes No	304	79.2
**Q#2**. If yes for Q#1, what are the clinical signs of the disease?	Coughing	10	3.28
	Coughing, Sneezing, Nasal discharge	1	0.32
	Coughing, Sneezing, Appetite loss, and fever	1	0.32
	Coughing, Nasal discharge, appetite loss, and fever	1	0.32
	Coughing, sneezing	6	1.97
	Coughing, Nasal discharge	17	7.02
	Coughing, appetite loss, and fever	1	0.32
	Sneezing	4	1.31
	Sneezing, Nasal discharge	7	2.30
	Sneezing, Difficult breathing	3	0.98
	Nasal discharge	15	4.93
	Nasal discharge, Difficult breathing	9	2.96
	Nasal discharge, Appetite loss, and Fever	8	2.63
	Difficult breathing	9	2.96
	Difficult breathing, Appetite loss, and Fever	3	0.98
	Appetite loss, and Fever	14	4.60
	Coughing, Sneezing, Nasal discharge, Difficult breathing	33	10.85
	I don't know	162	53.28
**Q#3**. Did you encounter pasteurellosis in your flock before?	Yes No	242	63
**Q#4**. If yes Q#3, which of the factors were associated with occurrence of pasteurellosis?	Transportation, Shortage of Feed and Water	1	0.41
	Transportation, Overcrowding	2	0.82
	Shortage of Feed and Water	32	13.22
	Shortage of Feed and Water, Overcrowding, Seasonal Variation	3	1.23
	Shortage of Feed and Water, Overcrowding	6	2.47
	Shortage of Feed and Water, Seasonal Variation	3	1.23
	Overcrowding	17	7.02
	Overcrowding, Seasonal Variation	1	0.41
	Overcrowding, Going to/coming from a Market	1	0.41
	Seasonal Variation	58	23.96
	Seasonal Variation, Going to/Coming from a Market	1	0.41
	Going to/coming from Market	8	3.30
	Transportation, Shortage of Feed and Water, Overcrowding, Seasonal Variation	13	5.37
	I don't know	88	36.36
The Composite Score of Knowledge on Ovine Pasteurellosis	Adequate Knowledge	106	27.6
	Inadequate Knowledge	278	72.4

**Table 2:** Respondents' attitude toward ovine pasteurellosis (*n* = 304).

**Attitude queries**	**Response**	**Frequency**	**Percentage**
**Q#1** Do you agree that ovine pasteurellosis is a concerning disease?	Strongly agree Agree Neither agree nor disagree Disagree Strongly disagree	47 74 88 78 17	15.46 24.34 28.94 25.65 5.59
**Q#2** Can spread of ovine pasteurellosis between sheep be prevented?	Strongly agree Agree Neither agree nor disagree Disagree Strongly disagree	62 109 100 21 12	20.39 35.8 5 32.89 6.90 3.94
**Q#3** Do you trust vaccination of animals against ovine pasteurellosis as a means of preventing the disease?	Strongly agree Agree Neither agree nor disagree Disagree Strongly disagree	77 89 100 22 16	25.32 29.27 32.89 7.23 5.26
**Q#4** Would you report sick or dead animals to the local authorities/veterinary officers?	Strongly agree Agree Neither agree nor disagree Disagree Strongly disagree	46 64 111 70 13	15.13 21.05 36.51 23.02 4.27
**Q#5** Do you think that health care providers can handle ovine pasteurellosis outbreaks very well?	Strongly agree Agree Neither agree nor disagree Disagree Strongly disagree	69 86 101 35 13	22.69 28.2 8 33.22 11.51 4.27
The Composite Score of Attitude on Ovine Pasteurellosis	Favorable attitude Unfavorable attitude	153 151	50.32 49.68

### Demographic Characteristics of Respondents

A total of 384 respondents were considered from four (4) districts with the following distribution: 96 (25%)—Merawi; 95 (24.74%)—Farta; 100 (26.04%)—Gondar zuriya; and 93 (24.22%)—Dabat. In this survey, the majority of the respondents were males (84.4%, 324/384). Age-wise, respondents were categorized into three (3) groups as: 19–36 years (20.3%; 78/384), 37–55 years (47.1%; 181/384); and above 55 years (32.6%; 125/384). A relatively high number of respondents (44.5%, 171/384) owned a flock of 5 to 10 sheep while 34.6% (133/384) and 20.8% (80/384) had an average flock size of 1–5 and > 10 sheep, respectively. Regarding the respondents' literacy level, 85.9% did not have formal education, 7 and 5.2% had completed primary school and secondary school, respectively, while the remaining 1.8% had a college diploma (Table 3).

**Table 3:** Practices of respondents relevant to ovine pasteurellosis (*n* = 304).

**Practice queries**	**Response**	**Frequency**	**Percentage**
1. Do you travel a long distance to get veterinary services or market access?	No Yes	245 59	80.6 19.4
2. Do you isolate sheep when they are sick from respiratory illness?	Yes No	35 269	11.5 88.5
3. Do you drench drugs to your animals when affected with ovine pasteurellosis?	Yes No	263 41	86.5 13.5
4. Do you mix up your sheep flock with others?	Yes No	45 259	14.8 85.2
5. Do you often go to nearby veterinary clinics when one or more sheep are sick?	Yes No	285 19	93.75 6.25
The Composite Score of Practice on Ovine Pasteurellosis	Good practice Poor practice	236 68	77.6 22.4

### Respondents' Knowledge, Attitude, and Practices Pertinent to Ovine Pasteurellosis

#### Assessment of Knowledge

Questions about the local name, seasonal occurrence, clinical signs, and predisposing factors of ovine pasteurellosis were used to assess knowledge. Of all respondents, most (79.2%) knew ovine pasteurellosis by the local name “*Gororsa”*, of which more than half (53.28%) were not able to mention any of the clinical signs of the disease. On the other hand, 63% of respondents claimed to have had the disease occurred in their flocks, of which 63.64% knew one or more of the environmental stressors such as transportation, confinement, and shortage of feed or water contributing to the occurrence of the disease. However, comprehensive understanding of the disease was low as most of the respondents (72.4%) had inadequate knowledge about ovine pasteurellosis as shown by the low knowledge composite score (Table 4). Univariable and multivariable logistic regression analyses were carried out to assess the associations of demographic variables with knowledge about the disease as presented in Tables 5, 6. Among these, age and flock size were selected for the multivariable model (*p* < 0.25). In the final model, age-groups: 37–55 years-old [OR: 2.92, 95% CI (1.43–5.98), *p* = *0.0*03] and >55 years-old [OR: 2.44, 95% CI (1.15–5.19), *p* = *0.0*2], had an adequate knowledge level about ovine pasteurellosis than the age-group 19–36 years-old. Similarly, having higher numbers of sheep (>10) was significantly associated with a good knowledge level [OR: 2.59, 95% CI (0.39–4.84), *p* = 0.003] about the disease (Table 6).

**Table 4:** Univariable analysis of putative risk factors in relation to serological status using mixed effect logistic regression models including village as a random effect.

**Putative factors**		**Tested samples**	**Seroprevalence (%)**	**Odds ratio (OR)**	**95% Confidence interval (CI)**	***p*-values**
Flock size	<5 sheep	111	84.68	Ref.		
	5–10					
	sheep	272	90.07	1.51	0.73–3.1	0.262
	>10 sheep	217	93.08	1.19	0.49–2.92	0.696
Sex	Male Female	116 484	79.31 92.77	Ref. 3.75	1.91–7.37	0.000
Age	<1 year 1–2 years >2 years	61 96 443	80.33 86.46 92.33	Ref. 2.82 6.88	0.93–8.55 2.54–18.63	0.066 0.000
Concurrent infection/disease	Yes No	253 347	91.3 89.34	Ref. 0.83	0.45–1.55	0.567
Contact with other flock	Yes No	515 26	90.99 76.47	Ref. 0.88	0.34–2.28	0.797
Animal husbandry	Extensive Semi-intensive Intensive	435 131 34	93.1 83.97 76.47	Ref. 0.398 0.88	0.45–2.12 0.31–2.48	0.971 0.803
Altitude	Midland Highland	400 200	85.75 99	Ref. 16.09	2.26–114.64	0.006

*Ref., reference group*.

**Table 5:** The final multivariable mixed-effect logistic regression model.

**Demographic factors**	**Category**	**Coefficients**	**Odds Ratio (OR)**	**95% Confidence interval (CI)**	***p-values***
Sex	Male		Ref.		
	Female		2.45	1.09–5.52	0.031
Altitude	Midland		Ref.		
	Highland		20.29	20.29	0.004

*Ref., reference group*.

**Table 6:** Socio-demographic characteristics of respondents (*n* = 384).

**Variables**	**Category**	**Frequency (%)**
Sex	Male Female	324 (84.4) 60 (15.6)
Age-groups (years)	19–36 37–55 >55	78 (20.3) 181 (47.1) 125 (32.6)
Educational status	No formal education Primary school Secondary school College diploma	330 (85.9) 27 (7) 20 (5.2) 7 (1.8)
Flock size (Number of sheep)	1–5 5–10 >10	133 (34.6) 171 (44.5) 80 (20.8)

#### Assessment of Attitude

Taking five (5) attitude assessing questions into consideration, the composite score of the respondents having a favorable attitude regarding ovine pasteurellosis was about 50.26% (Table 7). The association between the demographic variables and the attitude of participants toward the disease is presented in Table 8. Both univariable and multivariable logistic regression models were fitted for demonstrating the attitude about the disease. Only sex was found to likely affect the attitude of farmers in the final multivariable logistic regression model. Being a male [OR: 0.56, 95% CI (0.32–0.99), *p* = *0.046*] was significantly associated with having a favorable attitude toward the disease as compared to females.

**Table 7:** Association of demographic variables with knowledge level of participants about ovine pasteurellosis (*n* = 384).

**Demographic variables**	**Category (n)**	**Respondents with good knowledge level (%)**	**OR (95% CI)**	***p*-values**
Sex	Male (324) Female (60)	91 (28.09) 15 (25)	Ref. 0.85 (0.45–1.61)	0.624
Age-groups in year (n)	19–36 (78) 37–55 (181) >55 (125)	11 (14.1) 59 (32.6) 36 (28.8)	Ref 2.95 (1.45–5.99) 2.46 (1.17-−5.19)	0.003 0.018
Educational status	No education (330) Primary school (27) Secondary school (20) College diploma (7)	92 (27.88) 6 (22.22) 6 (30) 2 (28.57)	Ref. 0.74 (0.29–1.89) 1.11 (0.41–2.97) 1.03 (0.19–5.43)	0.528 0.838 0.968
Flock size (Number of sheep)	1–5 (133) 5–10 (171) >10 (80)	27 (20.3) 47 (27.49) 32 (40)	Ref. 1.49 (0.87–2.55) 2.62 (1.41–4.84)	0.149 0.002

*Ref., reference group. It is a group against which we compared the other exposure groups*.

**Table 8:** Final multivariable logistic regression model of factors associated with respondents' knowledge about ovine pastuerellosis (*n* = 384).

**Demographic variables**	**Category (*n*)**	**Coefficients**	**SE**	**OR (95% CI)**	***p*-values**
Age-groups in years (n)	19–36 (78) 37–55 (181) >55 (125)	2.94 2.33	1.07 0.94	Ref. 2.92 (1.43–5.98) 2.44 (1.15–5.19)	0.003 0.020
Flock size (No. of sheep)	1–5 (133) 5–10 (171) >10 (80)	1.36 0.003	0.41 3.00	Ref. 1.46 (0 0.85–2.52) 2.59 (0.39–4.84)	0.173 0.003

*Ref., reference group*.

#### Assessment of Practice

Taking five (5) practice assessing questions into consideration, the composite score of the respondents having a good practice level was about 77.6% (Table 9). About one-fifth (19.4%) of respondents had to walk their animals in long distances to search for market and veterinary services. In this survey, 93.8% of respondents had the practice of taking sick animals to veterinary clinics for treatment purposes. In addition to seeking veterinary services, 86.5% reported that they drench their sheep with drugs for various diseases including ovine pasteurellosis. Similarly, the majority (88.5%) of them did not practice isolation, i.e., they tend to manage healthy and sick sheep mixed up together. The association between socio-demographic factors and the practice of participants is presented in Table 10. Of all the factors, only age (age-group >55 years old) was found to have a significant association with good practice toward the disease [OR: 2.05, 95% CI (1.01–4.18), *p* = 0.048] in the final multivariable logistic regression model.

**Table 9:** Association of demographic variables with attitudes of respondents toward ovine pasteurellosis (*n* = 304).

**Demographic variables**	**Category (*n*)**	**Respondents with favorable attitudes**	**OR (95% CI)**	***p*-values**
Sex	Male (256) Female (48)	134 (52.34%) 18 (37.5%)	Ref. 0.56 (0.32–0.99)	0.046
Age-groups in year (*n*)	19–36 (62) 37–55 (143) >55 (99)	32 (51.6%) 77 (53.84%) 44 (44.4%)	Ref 1.12 (0.66–1.91) 0.75 (0.42–1.32)	0.672 0.312
Educational status	No formal education (261) Primary school (21) Secondary school (16) College diploma (6)	125 (47.89%) 13 (61.90%) 13 (81.25%) 2 (33.33%)	Ref. 1.85 (0.82–4.16) 4.35 (1.42–13.3) 0.44 (0.83–2.28)	0.137 0.010 0.325
Flock size (Number of sheep)	1–5 (105) 5–10 (136) >10 (63)	53 (50.47%) 63 (46.3) 37 (58.73)	Ref. 0.85 (0.54–1.33) 1.40 (0.80–2.46)	0.470 0.236

*Ref., reference group*.

**Table 10:** Association of demographic variables with practice of animal owners toward ovine pasteurellosis (*n* = 304).

**Demographic variables**	**Category (*n*)**	**Respondents with good practices**	**OR (95% CI)**	***p*-values**
Sex	Male (256) Female (48)	200 (78.12) 36 (75)	Ref 0.84 (0.44 −1.59)	0.599
Age-groups in years (*n*)	19–36 (62) 37–55 (143) >55 (99)	46 (74.19) 105 (73.42) 85 (85.85)	Ref 0.95 (0.52–1.75) 2.05 (1.01–4.18)	0.883 0.048
Educational status	No formal education (261) Primary school (21) Secondary school (16) College diploma (6)	204 (78.16) 15 (71.42) 11 (68.75) 6 (100)	Ref. 0.66 (0.82–4.16) 0.396 (1.42–13.3) -	0.352 0.396 -
Flock size (Number of sheep)	1–5 (105) 5–10 (136) >10 (63)	78 (74.28) 108 (79.41) 50 (79.36)	Ref. 1.29 (0.7–2.20) 1.37 (0.70–2.69)	0.355 0.355

*Ref., reference group*.

## Discussion

Ruminant production is one of the potential sectors identified by the livestock master plan of the Ethiopian government (LMP, 2015–2020) to achieve food security (39). However, this is constrained by multiple factors. Ovine pasteurellosis due to *M. haemolytica, B. trehalosi*, and *P. multocida*, is a priority infectious disease for the sheep production sector causing a substantial economic loss through high mortality and morbidity (6–9). Despite the current prevention and control attempts through vaccination, the problem remains to be a major challenge in Ethiopia including in the Amhara region (26).

In this study, we determined the seroprevalence and distribution of various serotypes of *M. haemolytica, P. multocida*, and *B. trehalosi* in the Western Amhara sub-region which informs the dominant serotypes for inclusion in a multivalent ovine Pasteurella vaccine. We also assessed the predisposing factors of ovine pasteurellosis which helps better understand and control the disease in the study area. In addition, we investigated the KAP of farmers/animal caretakers toward the disease which could enable identify and address major gaps for implementation of effective disease management strategies through a participatory approach.

The overall seroprevalence of ovine pasteurellosis in this study was 90.17% which is comparable to the 100 and 83% seroprevalence reported by Berhe et al. (11), and Sisay and Zerihun (8). This high prevalence is an indirect indication that the disease is negatively impacting sheep productivity as well as the livelihood of smallholder farmers which calls for urgent and effective interventions. Despite this, the study could not rule out if this enormous seroprevalence is due to recent infections or the cumulative effect of chronic exposure to the disease. Serotyping of Pasteurella species is vital to inform/dictate the composition of ovine Pasteurella vaccines that best suits the field situation (40). In the current study, we reported six (6) serotypes at different levels of occurrence as followed: A1 (39.17%), A2 (74.83%), A7 (77.83%), *P. multocida* serotype A (60%), T4 (62%), and T15 (64%). Higher or lower seroprevalence of distinct serotypes could be reported at varied frequencies in different areas (8, 11, 12, 17, 41–43) due to variation in sample size, laboratory methods used, management practices, level of environmental stressors, or exacerbating factors, climatic factors, and occurrence of concurrent pathogens (9). Different serotypes can be associated with a wide range of clinical diseases. Isolates of biotype A of *M. haemolytica* are associated with respiratory disease in sheep and septicaemia in young lambs, while biotype T isolates have been associated with septicaemia in young adult sheep. All serotypes can be involved in pneumonic pasteurellosis in sheep, but serotype A2 is the most commonly isolated serotype from cases of ovine pneumonic pasteurellosis (17). Serotypes of *B. trehalosi* are most commonly associated with septicemia and pneumonic pasteurellosis in sheep <2-months-old (44). Young lambs are reported to be affected by polyarthritis due to *P. multocida* (44). The regular use of mono-serotype vaccine composed of inactivated *P. multocida* biotype A vaccine (9) in areas intricate with widespread distribution of various serotypes that lack cross-protection (23, 40) may explain why the disease remains a challenge with the current vaccination practices. In addition, co-infection with two or more serotypes on a single animal has been well recorded in almost all sampled (90.17%) animals. Similar findings have been reported by (11, 45) in Northern Ethiopia. Whether multiple serotype infection exhibits a different clinical or immunological outcome as opposed to mono-serotype infections is an area of further investigation.

In this study, the sero-prevalence of Pasteurella was found to be higher in female animals than in males [OR: 2.45, 95% CI (1.09–5.52), *p* = 0.031] which does not comply with the findings of other studies that observed no association between Pasteurella infection and sex (11, 12, 46). However, it has been reported that female animals are at high risk of succumbing to Pasteurella infections and other diseases during gestation, lambing, and lactation (47). In addition to sex, altitude was shown to influence the prevalence of the disease with high altitude favoring the occurrence of the latter which could be due to the windy, cold and unstable nature of the weather. Nevertheless, flock size, age, husbandry system, and maternal status did not show a statistically significant association with seropositive.

In the questionnaire survey, the proportion of animal owners who heard about ovine pasteurellosis was high (79.2%). Similarly, Mengstie et al. (48) reported that 81% of respondents in their study were aware of ovine pasteurellosis. Given the ubiquity of the disease, and the country having the largest sheep population in Africa, it is likely that most sheep owners could hear/learn about the disease at some point in their life. However, only 46.72% were able to list one or more of the clinical signs of the disease. Similarly, the proportion of respondents who claimed to encounter the disease in their flock was also relatively higher (63%), of which 63.63% were aware that the disease is initiated in the presence of one or more of the stress factors, i.e., mingling with other flocks in market areas, transportation, feed and water shortage, overcrowding, and seasonal variations. Seasonal variations associated with feed availability and weather fluctuation have been reported to drive the occurrence of diseases such as pasteurellosis (47). Collectively, the comprehensive understanding of sheep owners about ovine pasteurellosis was inadequate as indicated by the low composite knowledge score of the majority (74.2%). In this study, respondents age-group 37–55 years old [OR: 2.92, 95% CI (1.43–5.98), *p* = 0.003] and >55 years old [OR: 2.44, 95% CI (1.15–5.19) *p* = 0.02] had a good knowledge level about ovine pasteurellosis than age-group, 19–35 years old, which is likely to be associated with the make-up of the families largely comprising adults older than 35 years who may have more experience with managing animals. Likewise, farmers having a relatively higher number of sheep (>10) had a good knowledge level [OR: 2.59, 95% CI (0.39–4.84), *p* = 0.003] about the disease. Ovine pasteurellosis is favored by overcrowding which induces stress, compromising the immunity of animals thus creating an ideal condition for the occurrence of the disease (47). Large flock size is susceptible to the disease unless sufficiently managed (which is the case with these farmers having less space), thus, frequent encounters with the disease could have increased owners' awareness about the latter.

In this study, a comparable number (50.26%) of participants had a favorable attitude toward ovine pasteurellosis as shown by positive answers on reporting the disease to local veterinary experts, the importance of the disease, its transmissibility, and vaccination of animals as a means to combat it. The fact that farmers trusted vaccination as a means to control and prevent the disease is expected to increase vaccine uptake thus increasing vaccination coverage in the area.

Of the socio-demographic factors, only sex was found to have a significant association with attitude [OR: 0.56, 95% CI (0.32–0.99) *p* = 0.046]. Being a male is associated with having a favorable attitude toward the disease. This could result from the presence of a larger number of male respondents than females in the questionnaire (84.21%). Nonetheless, it can be anticipated that males are most likely to manage or monitor sheep flocks both in-door as well as in the field which could increase the level of awareness of the disease. In addition, they are the family members that manage to participate in various training and professionals-led community discussions relevant to animal disease. In this study, 77.6% of the respondents followed good practices in relation to ovine pasteurellosis (as shown by the composite practice score). Nevertheless, the majority (86.5%) of them attempt to treat diseased sheep by themselves at their home in addition to seeking veterinary services. Administration of antibiotics by un-professionals is malpractice highly linked to antimicrobial resistance (49, 50) and drug toxicity (51) which requires prompt intervention. Similarly, the majority (88.5%) of the farmers did not practice animal isolation, i.e., they tend to manage healthy and sick sheep mixed up together.

From the demographic variables, only age (>55 years old) was found to seemingly influence practice toward the disease [OR: 2.05, 95% CI (1.01–4.18), *p* = *0.048*]. In the study areas, activities such as consultation with veterinarians /animal health officers, participation in awareness-raising programs, and engagement in disease management are likely to be handled by old family members.

## Conclusion

This study showed that ovine pasteurellosis is an important disease of sheep in the study area. An overall seroprevalence of 90.17% was reported with widespread distribution of six (6) serotypes, i.e., A1 (39.17%), A2 (74.83%), A7 (77.83%), *P. multocida* serotype A (60%), T4 (62%), and T15 (64%). The existence of such diverse serotypes that exhibited little-to-none cross-protection in the study areas where the mono-serotype vaccine is regularly used, is likely to provide no adequate protection against the disease. The judgment of the inclusion of one or more serotypes should be dictated by composite criteria that involve the abundance of individual serotypes, the clinical outcome, and the resultant economic impact. In addition, this study reported the presence of multiple serotype co-infection in the majority (90.17%) of the study animals. The clinical or immunological implication of mixed serotypes co-infection as compared to mono-serotype infection is a subject of further studies. Assessment of risk factors indicated that sex and agro-ecological zones are likely to affect the prevalence of the disease. Female animals need to be managed with precaution particularly during physiologically stressing conditions such as pregnancy, lambing, and lactation to avoid the incidence of the disease. Similarly, flocks in highland areas may require appropriate and timely management during and prior to seasons of weather fluctuation to prevent outbreaks of the disease. Moreover, this study highlighted that farmers in the study areas had low levels of knowledge about key aspects of ovine pasteurellosis. This in turn may call for the provision of various awareness-raising training to improve the knowledge, attitude, and practice of farmers toward the disease for implementation of effective disease control strategy through a participatory approach engaging sheep owners.

## Data Availability Statement

The original contributions presented in the study are included in the article/Supplementary Material, further inquiries can be directed to the corresponding author.

## Ethics Statement

The animal study was reviewed and approved by Institutional Review Board of University of Gondar. Written informed consent was obtained from the owners for the participation of their animals in this study.

## Author Contributions

SI responsible for the conception of the study and prepared the initial draft of the manuscript. KG, BA, and HG collected the blood samples and the questionnaire data. WM, SM, and KG analyzed the data. SI, WM, SM, BM, and AA supervised the conduct of the study. All authors have read, revised, improved, and approved the final manuscript. All authors contributed to the article and approved the submitted version.

## Funding

This work is part of a mega research project funded by the University of Gondar, the office of the vice president for research and community service (Budget code: VPRCS 6223, 2019/20-2020/21) to SI as PI and the co-authors as co-I of the project. We declare that the funding body did not contribute to the execution of any part of the study, interpretation of the results, and write-up of this article.

## Conflict of Interest

The authors declare that the research was conducted in the absence of any commercial or financial relationships that could be construed as a potential conflict of interest.

## Publisher's Note

All claims expressed in this article are solely those of the authors and do not necessarily represent those of their affiliated organizations, or those of the publisher, the editors and the reviewers. Any product that may be evaluated in this article, or claim that may be made by its manufacturer, is not guaranteed or endorsed by the publisher.
