# Supplementary material for: Serotyping and Seroprevalence of Mannheimia haemolytica, Pasteurella multocida, and Bibersteinia trehalosi and Assessment of Determinants of Ovine Pasteurellosis in West Amhara Sub-region, Ethiopia
Source: Front Vet Sci. 2022 May 19;9:866206. doi: 10.3389/fvets.2022.866206 (PMC9161746; doi:10.3389/fvets.2022.866206)
Supplement: Supplementary file 1 [file Table_1.DOCX]

**Supplementary Material 1.** Questions Used to Assess Risk Factors of Ovine Pasteurellosis and Knowledge, Attitude, and Practice of Farmers towards the Disease in West Amhara sub-region, Ethiopia

| S.N | **Part I: Sociodemographic Characteristics of Respondents** |
| --- | --- |
| 1 | Sex: Male  Female |
| 2 | Age-group: 19-36 years-old  37-55 years-old  >55 years-old |
| 3 | Educational Status: No formal education  1º school  2 º school  College diploma |
| **Part II: Putative Risk Factors** | |
| 1 | Flock size: 1-5 sheep  5-10 sheep  >10 sheep |
| 2 | Sex: Male  No |
| 3 | Age: < 1 year-old  1-2 years-old  >2years-old |
| 4 | Concurrent infection/disease: Yes  No |
| 5 | Contact with other flocks: Yes  No |
| 6 | Animal husbandry system: Extensive  Semi-intensive  Intensive |
| 7 | Altitude: Midland  Highland |
|  | **Part-III: Knowledge, attitude, and Practice questions** |
|  | **Knowledge Section** |
| 1 | Have you ever heard of ovine pasteurellosis (*‘Gororsa’*)? Yes  No |
| 2 | If yes for Q#1, what were the clinical signs you observed? ________, __________, __________, __________, __________ |
| 3 | Did you encounter pasteurellosis in your flock before? Yes  No |
| 4 | If yes for Q#3, which of the factors were associated with occurrence of pasteurellosis? __________, __________, __________, __________ |
|  | **Attitude Section** |
| 1 | Do you agree that ovine pasteurellosis is a concerning disease? Yes  No |
| 2 | Can spread of ovine pasteurellosis between sheep be prevented? Yes  No |
| 3 | Do you trust vaccination of animals against ovine pasteurellosis  as a means of preventing the disease? Yes  No |
| 4 | Would you report sick or dead animals to local authorities/veterinary officers? Yes  No |
| 5 | Do you think that animal health care providers  can handle ovine pasteurellosis outbreaks very well? Yes  No |
|  | **Practice Section** |
| 1 | Do you travel long distance to get veterinary services or market access? Yes  No |
| 2 | Do you isolate sheep when they are sick from respiratory illness? Yes  No |
| 3 | Do you drench drugs to your affected when affected with ovine pasteurellosis? Yes  No |
| 4 | Do you mix up your sheep flock with others? Yes  No |
| 5 | Do you often go to nearby veterinary clinics when one or more sheep are sick? Yes  No |
